# Live cell monitoring of double strand breaks in *S*. *cerevisiae*

**DOI:** 10.1371/journal.pgen.1008001

**Published:** 2019-03-01

**Authors:** David P. Waterman, Felix Zhou, Kevin Li, Cheng-Sheng Lee, Michael Tsabar, Vinay V. Eapen, Allison Mazzella, James E. Haber

**Affiliations:** 1 Department of Biology, Brandeis University, Waltham, Massachusetts, United States of America; 2 Rosenstiel Basic Medical Sciences Research Center, Brandeis University, Waltham, Massachusetts, United States of America; UC Davis, UNITED STATES

## Abstract

We have used two different live-cell fluorescent protein markers to monitor the formation and localization of double-strand breaks (DSBs) in budding yeast. Using GFP derivatives of the Rad51 recombination protein or the Ddc2 checkpoint protein, we find that cells with three site-specific DSBs, on different chromosomes, usually display 2 or 3 foci that may coalesce and dissociate. This motion is independent of Rad52 and microtubules. Rad51-GFP, by itself, is unable to repair DSBs by homologous recombination in mitotic cells, but is able to form foci and allow repair when heterozygous with a wild type Rad51 protein. The kinetics of formation and disappearance of a Rad51-GFP focus parallels the completion of site-specific DSB repair. However, Rad51-GFP is proficient during meiosis when homozygous, similar to *rad51* “site II” mutants that can bind single-stranded DNA but not complete strand exchange. Rad52-RFP and Rad51-GFP co-localize to the same DSB, but a significant minority of foci have Rad51-GFP without visible Rad52-RFP. We conclude that co-localization of foci in cells with 3 DSBs does not represent formation of a homologous recombination “repair center,” as the same distribution of Ddc2-GFP foci was found in the absence of the Rad52 protein.

## Author summary

Double strand breaks (DSBs) pose the greatest threat to the fidelity of an organism’s genome. While much work has been done on the mechanisms of DSB repair, the arrangement and interaction of multiple DSBs within a single cell remain unclear. Using two live-cell fluorescent DSB markers, we show that cells with 3 site-specific DSBs usually form 2 or 3 foci that can may coalesce into fewer foci but also dissociate. The aggregation and mobility of DSBs into a single focus does not depend on the Rad52 recombination protein that is required for various mechanisms of homologous recombination, suggesting that merging of DSBs does not reflect formation of a homologous recombination repair center.

## Introduction

The process of repairing a chromosomal double-strand break by Rad51- and Rad52-mediated homologous recombination in budding yeast has been defined by a combination of *in vitro* analysis of purified recombination proteins [1–3] and from “*in vivo* biochemistry” analyses of the kinetics of repair of site-specific DSBs [4]. Cleaved DNA ends are attacked by several 5’ to 3’ exonucleases to produce long 3’-ended single-strand DNA (ssDNA) tails, which are initially coated by the single-strand binding complex, RPA [5, 6]. RPA is displaced by Rad51 recombinase through the action of mediator proteins, including Rad52, creating a nucleoprotein filament composed primarily of Rad51 but also its paralogs, the Rad55-Rad57 heterodimer [7–9]. The Rad51 filament engages in a genome-wide search for a homologous sequence that could be on a sister chromatid, a homologous chromosome or at an ectopic location. Once the donor sequence is encountered, Rad51 catalyzes strand exchange to form a D-loop intermediate, the initial step in repair. The 3’ end of the invading strand then acts as a primer to initiate new DNA synthesis that leads to repair of the DSB via several pathways including gene conversion via synthesis-dependent strand annealing or by a double Holliday junction pathway [4]. A combination of Southern blot, PCR and chromatin immunoprecipitation (ChIP) experiments have shown that DSB repair proceeds by a series of kinetically slow steps, taking more than an hour to complete (reviewed in [4]).

In haploid cells, successful recombination between a nuclease cleaved site with an ectopic homologous donor sequence is strongly dictated by the prior proximity of the donor with the region in which the cleavage site has been inserted [10–13], where proximity was determined by their contact probability of sequences, as measured by chromosome conformation capture methods [14–16]. The creation of a DSB results in increased chromatin movement, which may increase the likelihood of contact between two loci [12, 17–22]. Single particle tracking of fluorescently tagged loci adjacent to DSBs has shown that repair through homologous recombination causes an increase in chromatin movement dependent on the number of DSBs present [17, 18, 22]. This increased movement in response to DSBs has been shown to be dependent on the DNA damage checkpoint [12, 18], DNA repair factors [17, 18] and chromatin remodelers [12]. Recently, a role for microtubules in controlling chromatin mobility after DNA damage in budding yeast has been proposed [20, 23]; but others have found DSB-associated movement to be independent of microtubules [24]. There is also evidence that nuclear actin, in association with the chromatin remodeler, Ino80, may also play a role in chromatin dynamics [25]. When the ends of the DSB fail to encounter a homologous donor sequence, or when there is no donor, an unrepaired break eventually enters a different pathway, where it associates with the nuclear envelope through its association with the spindle-pole body and nuclear envelope protein Mps3 [26, 27] and to the nuclear pore [28].

One approach to the study of DSB repair in budding yeast has been the use of live and fixed-cell microscopy to monitor the behavior of different fluorescently tagged repair-associated proteins [29]. The most thoroughly studied is Rad52, the key mediator for the assembly of the Rad51 filament, but which is also critical in later strand-annealing steps [30]. Strikingly, when there are multiple DSBs, created by ionizing radiation or by site-specific endonucleases, there often appears to be a single fluorescent Rad52 focus. The recruitment of more than one DSB to a common focus has also been studied by creating fluorescent chromosome tags (arrays of LacO or TetO sequences) near different DSBs. Lisby *et al*. [29] found that DSBs created by two different site-specific endonucleases co-localized in about 50% of haploid cells. These observations have led to the idea that there could be a “repair center” where recombination proteins might accumulate to facilitate DSB repair [29]. However, immunofluorescent staining of spread nuclei with multiple DSBs found that the number of foci directly correlated with the number of DSBs [31, 32].

To directly test the requirement for Rad52 in organizing DSBs, we monitored the localization of the Rad52-indpendent DSB binding protein, Ddc2, yeast’s homolog of the mammalian ATRIP protein that has been previously shown to bind near a DSB and to recruit Mec1^ATR^ kinase [33, 34]. With Ddc2-GFP, we show that cells which have three site-specific DSBs form multiple, highly dynamic foci that often coalesce and separate, but most cells do not form a single fluorescent focus. The number of foci and their motion are independent of Rad52 and microtubules.

We also constructed and characterized a Rad51-GFP fusion protein. Previously, a Rad51-GFP fusion was characterized in *Arabidopsis*, where it proved to be defective in mitotic DSB repair, but competent in meiosis [35]. This phenotype resembles the “site II” mutation of *Saccharomyces cerevisiae* Rad51, which can bind ssDNA but prevents ternary complex formation with Rad51 bound to ssDNA and thus fails to complete strand invasion and DSB repair in mitotic cells [36, 37]. Similar results were obtained using an isoform of Rad51-GFP from rice and humans *in vitro* [38]. In fission yeast, Rad51’s homolog Rhp51, when fused with CFP, proved to be UV-sensitive and incapable of carrying out repair on its own, but this defect was complimented by expression of wild type Rhp51 [39]. In budding yeast a YFP-Rad51 fusion forms DSB-dependent foci despite its inability to participate in HR in mitotic cells [40]. This loss of function was suppressed by introducing a gain-of-function Rad51-I345T mutation, which largely restored viability upon irradiation [41]. Here we show that budding yeast Rad51-GFP binds to site-specific DSBs in mitotic cells but cannot catalyze homologous recombination when it is the only allele present; however, unlike in *Arabidopsis*, it is not dominant-negative [35]. Consequently, Rad51-GFP can be used to follow fluorescently-labeled filaments that are engaged in functional recombination.

When using Rad51-GFP to examine localizations of 3 site-specific DSBs, we found the distribution of foci to be nearly identical to the distribution found with Ddc2. When Rad51-GFP and Rad52-RFP foci are co-expressed, they co-localize to multiple DSBs, although some limitation in Rad52-RFP expression or a propensity for self-aggregation appears to restrict the number of Rad52 foci.

## Materials and methods

### Strain and plasmid construction

Standard yeast genome manipulation procedures were used for all strain constructions [42]. Linear DNA and plasmids were introduced by the standard lithium acetate transformation procedure [43]. To C-terminally tag Rad51 and Ddc2 with eGFP, PCR primers were used to amplify the eGFP fragment from pFA6a-GFP(S65T) and the *TRP1* or KAN selectable marker in the Longtine collection [44] and introduced to the appropriate parent strain by lithium acetate transformation. To create monomeric emGFP, alanine 206 was mutated to lysine by single-stranded template repair using Cas9 as previously described [45]. Briefly, oligos DW549 and DW550 were duplexed and ligated into plasmid bRA89 after digestion with BplI to create plasmid pDW54. The plasmid together with oligo DW548 were co-transformed and selected for by plating YPD + hygromycin B. Isolates were screened for the correct insertion by PCR and sequencing. Strain genotypes are listed in (S1 Table). Primer sequences are listed in (S2 Table). Plasmids are listed in (S3 Table). Strain YCSL004 [46] carries three HO cleavage sites, at *MAT* (position 200 kb) on chromosome 3, at position 98 kb on chromosome 6 and position 252 kb on chromosome 2. These sites are located approximately 100, 50 and 15 kb from their respective centromeres.

### Growth conditions

To visualize the chromosomally integrated fluorescent tags (Rad51-GFP and Ddc2-GFP) after DNA damage, cells from a single colony were grown overnight in 5ml YEP + 3% lactic acid (YPLac). Cells were diluted to OD_600_ = 0.2 and grown for 4 h in 5 ml of fresh YPLac before addition of galactose to a final concentration of 2% to induce *GAL*::*HO* expression. For experiments that require the retention of an autonomously replicating plasmid, the same growth procedure except that cells were grown in SD-leucine media supplemented with 2% raffinose. For nocodazole treatment experiments, cells were first exposed to 2% galactose for 3 h then treated with 15 μg/ml nocodazole in DMSO for 10 minutes or the equivalent volume of only DMSO, and then imaged using the protocol detailed below.

### Plating assays and viability

The efficiency of DSB repair by homologous recombination was determined as described previously for strain YJK17 [47]. Briefly, cells were selected from a single colony on YPD plates and grown overnight in 5 ml of YPLac. Cells were diluted to OD_600_ = 0.2 and allowed to grow until OD_600_ = 0.5–1.0. Approximately 100 cells from each culture were then plated on YPGal (2% v/v) and YPD in triplicate and incubated at 30°C. Viability was calculated by dividing the number of colonies on YPGal by the number of colonies on YPD.

Adaptation assays in strain JKM179 were performed as previously described [48]. Briefly, cells were grown in YPLac or SD- media supplemented with 2% raffinose overnight then individual unbudded (G1) cells were plated on YPGal and observed microscopically for 24 h to determine the percent that remained arrested in the G2/M stage of the cell cycle.

Viability on MMS media was determined by as described previously [49]. Cells of the appropriate strain were selected from a single colony on YPD plates and grown overnight in 5 ml of selective media to near saturation. The following day, cultures were diluted to OD_600_ = 0.2 and left to grow at 30°C for 3–5 doublings. Cells were then diluted in 200 μl sterile water to OD_600_ = 0.2 in a 96-well plate and subsequently 10-fold serially diluted six times. Cell dilutions were then plated on YPD, -leu, and -leu +0.002% MMS plates and left to grow at 30°C for three days.

### Image acquisition and analysis

Prior to imaging, cells were washed twice in imaging media SC supplemented with 2% galactose or 2% raffinose and mounted on a glass depression slide coated with agarose supplemented with all amino acids. GFP, RFP and mCherry signals were visualized on a Zeiss AxioObserver spinning disk microscope with a 63x objective and an Andor Revolution spinning disk system consisting of a Nikon Ni-E upright microscope, equipped with a 100X (n.a. 1.45) oil immersion objectives, a Yokogawa CSU-W1 spinning disk head, and an Andor iXon 897U EMCCD camera. 10–12 z-stack images spaced at 0.5 μm were taken for each image. For live-cell time courses, z-stacked spaced at 0.5 μm were taken every 10 – 60s as indicated. Z-stacks were imported into FIJI and max-projected for image presentation or sum projected for foci intensity quantification. Foci were counted by adjusting the image color threshold to the average nuclear signal intensity for a given image and counting spherical regions that gave pixel intensity above the threshold. Foci and nuclear intensities were quantified by measuring the integrated intensities of circular regions from sum-projecting relevant z-stack slices. For colocalization analysis, z-stacks were imported into FIJI and split into the red and green channels. For each image, individual cells were selected and single corresponding z-stacks from the green and red channel were duplicated for analysis. The nucleus was selected as the region of interest ROI and the signal outside the ROI was cleared using the Clear Outside function in FIJI. To isolate the GFP and mCherry foci in the nucleus, the mean signal in the ROI was subtracted from the total nucleus signal. The plugin JACoP was used to calculate the Pearson’s Correlation Coefficient between the red and green channels.

### Chromatin immunoprecipitation

Chromatin immunoprecipitation (ChIP) was carried out as described previously [37]. In brief, cells were harvested from log-phase population. 45 ml of culture were fixed and crosslinked with 1% formaldehyde for 10 minutes after which 2.5 ml of 2.5 M glycine was added for 5 minutes to quench the reaction. Cells were pelleted and washed 3 times with 4°C TBS. Cell walls were disrupted by 1 min bead beating in lysis buffer, after which cells sonicated for 2 minutes. Debris was then pelleted and discarded, and equal volume of lysate was immunoprecipitated using α-ScRad51 antibody for 1 hour in 4°C, followed by addition of protein-A agarose beads for 1 h at 4°C. The immunoprecipitate was then salt washed 5 times, and crosslinking was reversed at 65°C overnight followed by proteinase-K addition for 2 h. Protein and nucleic acids where separated by phenol extraction. Chromatin association with Rad51 was assessed by qPCR. Individual timepoints were normalized to the antibody binding efficiency as determined by immunoprecipitation of Rad51 or Rad51-GFP from clarified whole cell extracts prepared by bead beating. α-ScRad51 antibodies were generous gifts from Akira Shinohara (University of Osaka, Osaka, Japan) and from Douglas Bishop (University of Chicago, Chicago, IL).

### DSB repair analysis by qPCR

Monitoring repair kinetics by qPCR was performed as described previously [50]. Single colonies were inoculated in 5 ml of media lacking leucine with 2% dextrose and grown overnight at 30°C. Overnight cultures were then diluted into 600 ml of YPLac and grown into log phase. DSBs were induced by adding 20% galactose to a final concentration of 2%. To track the dynamics of DSB repair 50 ml aliquots of each culture was collected every hour over 9 h. DNA was isolated using a MasterPure^TM^ Yeast DNA Purification Kit (Epicentre cat. MPY80200). The repair product, *MAT***a**-inc, was amplified using primers MATp13 and MATYp4 with a SYBR Green Master Mix using a Qiagen Rotor-Gene Q real-time PCR machine. To quantify the relative amount of *MAT***a**-inc in each sample, *SLX4* was used as a reference gene and was amplified using primers NS047-Slx4p7 and Slx4p1. Primer sequences are shown in (S2 Table).

## Results

### Live cell detection of DSBs with Ddc2-GFP with multiple DSBs

Ddc2 localizes to a broken DNA end, either directly or by binding to RPA [33, 34, 51] and previous studies have shown strong localization of Ddc2-GFP at DSB sites [52–54]. Cells suffering a single DSB arrest for 9–12 h, dependent on Ddc2, but then switch off the checkpoint and adapt to damage without completing DNA repair [55–57]. Cells lacking Ddc2, like those lacking its partner, Mec1, fail to arrest in response to a single DSB created by a galactose-inducible HO endonuclease in strain JKM179, where homologous recombination has been eliminated [58]. Appending eGFP (GFP-S65T) to the C-terminus of Ddc2 did not alter cell cycle arrest or adaptation following a DSB, indicating that the GFP moiety does not inhibit Ddc2’s checkpoint function (S1E Fig), confirming an earlier report [53].

**Fig 1:**
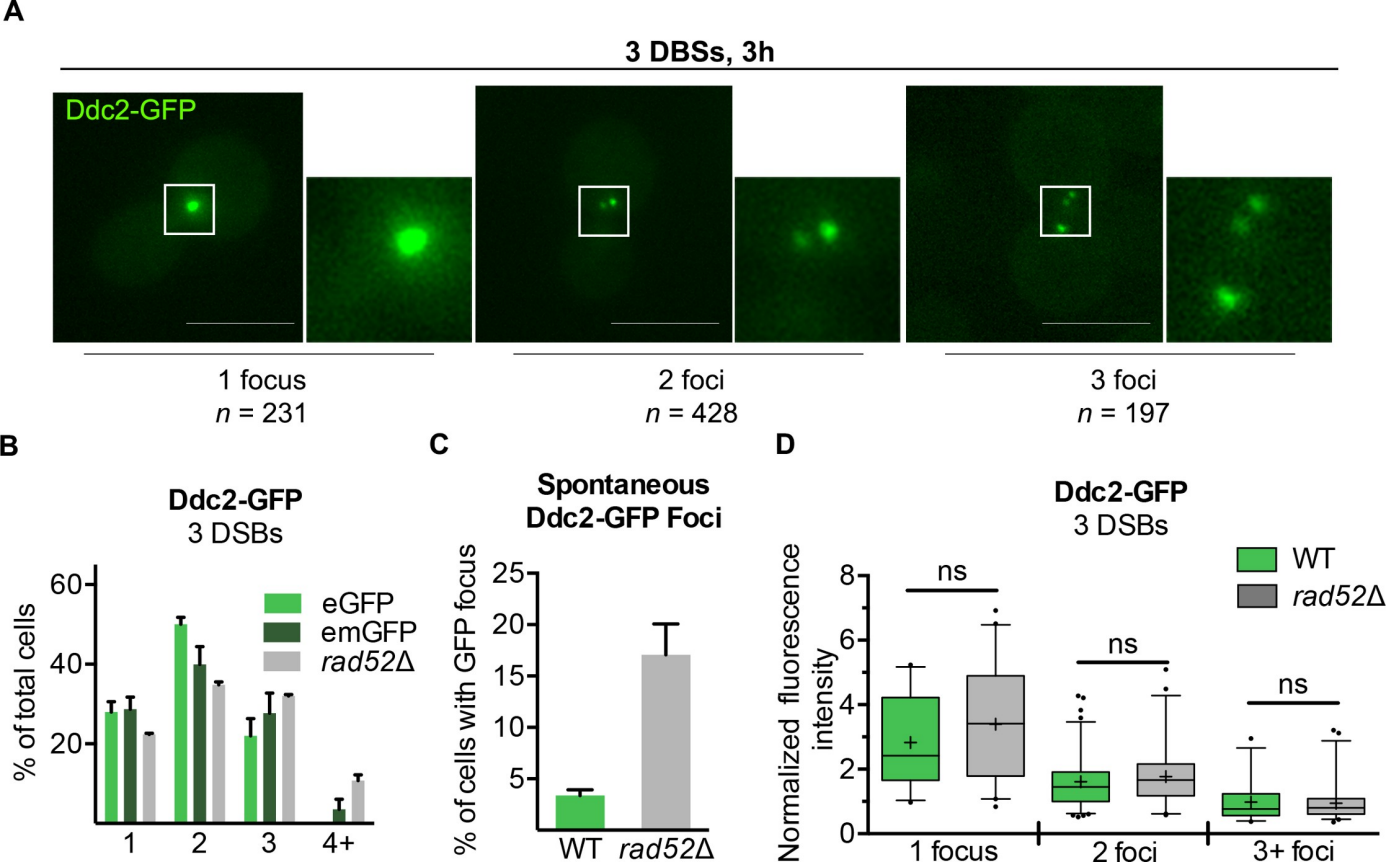
Analysis of Ddc2-GFP focus formation in strains with 3 DSBs. **A)** Representative images of 1, 2, or 3 foci in strain VE290 expressing endogenous Ddc2-eGFP 3 h after HO induction. *n* = total number of cells displaying the indicated number of foci from three biological replicates. Maximum projection of 10–12 z-stack images every 0.5 μm. Scale bar = 5 μm. **B)** Quantification of Ddc2-eGFP or Ddc2-emGFP foci in strain VE290 (WT) DW546 (*rad52*Δ) 3 h after HO induction. Error bars represent the SD of three biological replicates totaling >150 cells analyzed per experiment. **C)** Percentage of cells displaying at least one Ddc2-GFP focus in cycling WT or *rad52*Δ cells. Error bars represent the SD of three biological replicates totaling >150 cells analyzed per experiment. **D)** Background-subtracted fluorescence intensities of individual foci in strains VE290 (WT) and DW546 (*rad52*Δ) 3 h after HO induction as described in (B). Box plots display the median (black bar), mean (+), 25^th^ and 75^th^ percentiles (box ranges), 5th and 95^th^ percentiles (whiskers), and outliers (dots).

We monitored the localization of Ddc2-eGFP in strain YCSL004, where three HO cleavage sites on different chromosomes are each efficiently cut within 60 min of HO expression [46]. Three h after HO induction, we observed cells with 1, 2, or 3 foci with an average of 2 foci per cell (Figs 1A and 1B and S1A). Because the eGFP moiety is known to occasionally form dimers [59–61], which could promote colocalization of DSBs, we repeated our analysis in the monomeric eGFP-A206K (herein, emGFP) [59]. The proportion of cells with a single focus remain unaltered, although there was a larger percentage of cells containing 3 foci and fewer cells with 2 foci (Fig 1B). This distribution was unchanged in a *rad52*Δ derivative (Figs 1B and S1B).

In both wild type and *rad52*Δ cells suffering three DSBs, a small portion of cells contained more than 3 foci (WT = 3.5%, *rad52*Δ = 10.7%); rarely, even 5 foci were evident (Figs 1B and S1B). In cycling cells without HO-induced DNA damage, Ddc2-GFP foci were apparent in 4.9% of wild type cells and 17% of *rad52*Δ (Figs 1C and S1C). We conclude that cells which display more than 3 foci after HO induction likely reflect unrepaired spontaneous DNA damage arising during replication and independent of HO induction.

Even in the absence of Rad52, ~25% of cells displayed a single focus. It is evident from Fig 1A that the intensity of the single focus was much greater than the average intensities of each focus in cells displaying 2 or 3 foci. By measuring the fluorescence intensities of individual Ddc2-emGFP foci we determined that the signal intensity of the single focus in cells with one focus is equal to the sum of the signal intensities of 3 individual foci (Fig 1D). Thus, cells with a single focus likely have 3 DSBs that are co-localized. These intensities were unchanged in *rad52*Δ (Fig 1D).

### DSB foci are dynamic

**Fig 2:**
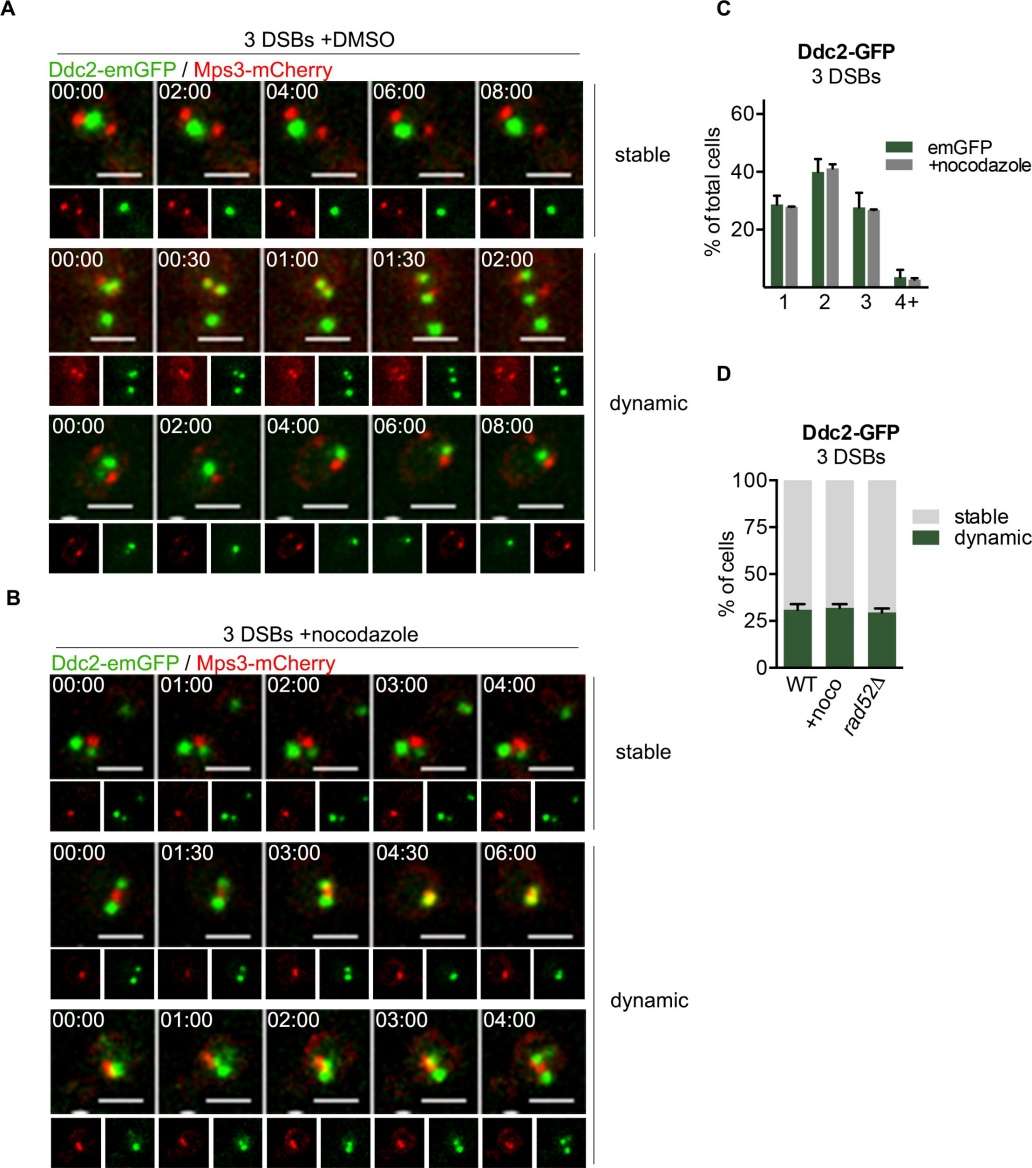
DSBs are dynamic. **A)** Single images of Ddc2-emGFP and Mps3-mCherry 3 h after HO induction in a 3 DSB strain. Scale bar = 2 μm. Time after start of image capture displayed. **B)** Identical to (A), but after treatment with 15 μg/ml nocodazole for 10 minutes before imaging. **C)** Ddc2-emGFP focus abundance in cells treated with DMSO (vehicle) or nocodazole, 3 h after galactose addition. **D)** Percentage of stable vs dynamic DSBs observed in (A) and (B). Data representative of three individual experiments examining >200 cells each.

Chromosomal mobility and chromatin persistence length are radically altered after the induction of a DSB [20, 62–64]. We examined the stability of foci with 3 DSBs by observing cells over a ten-minute period, 3 h after HO induction, using spinning disk confocal microscopy. 75% of cells at this time displayed large buds indicative of G_2_/M arrest. In ~70% of cells with large buds, the number of foci in a given cell remained constant over 10 min (Fig 2A and S4–S7 Movies). However, in ~30% of cells, the number and position of Ddc2-GFP foci were highly dynamic: the number of foci sometimes diminished, from three to two, or from two to one (Fig 2A). In other examples, a single focus split into two or three foci (Fig 2A and S8–S14 Movies). This behavior was unchanged in *rad5*2Δ (Fig 2D), with the exception that a few cells displayed >3 foci, as described above. We conclude that DSBs are dynamic and can coalesce or dissociate in a Rad52-independent fashion.

Microtubules have previously been implicated controlling chromatin dynamics in budding yeast [65, 66] and recent evidence has directly implicated microtubules in controlling localization and movement of DSBs [20, 23]. However, others have found that microtubules had no effect on DSB movement [24]. We tested whether the association of DSBs in our system was dependent on microtubules by treating cells with nocodazole 3 h after HO induction and monitoring Ddc2-emGFP foci. As a landmark, we included the spindle pole body (SPB)-associated Msp3-mCherry [67, 68]. Before nocodazole treatment, Mps3-mCherry was frequently localized to two well-separated foci, indicative of the position of SPBs in cells arrested prior to anaphase (Figs 2A and S2A); however, 10 min after nocodazole addition, the Mps3-mCherry puncta collapsed into a single dot, or two very closely spaced dots, as expected [69] (Figs 2B and S2B). Despite the absence of microtubules, the Ddc2-emGFP foci distribution and dynamics were unaltered (Fig 2B–2D). Therefore, the coalescence and separation of HO-induced DSBs is apparently independent of microtubules.

### Rad51-GFP forms a DNA damage-dependent focus

**Fig 3:**
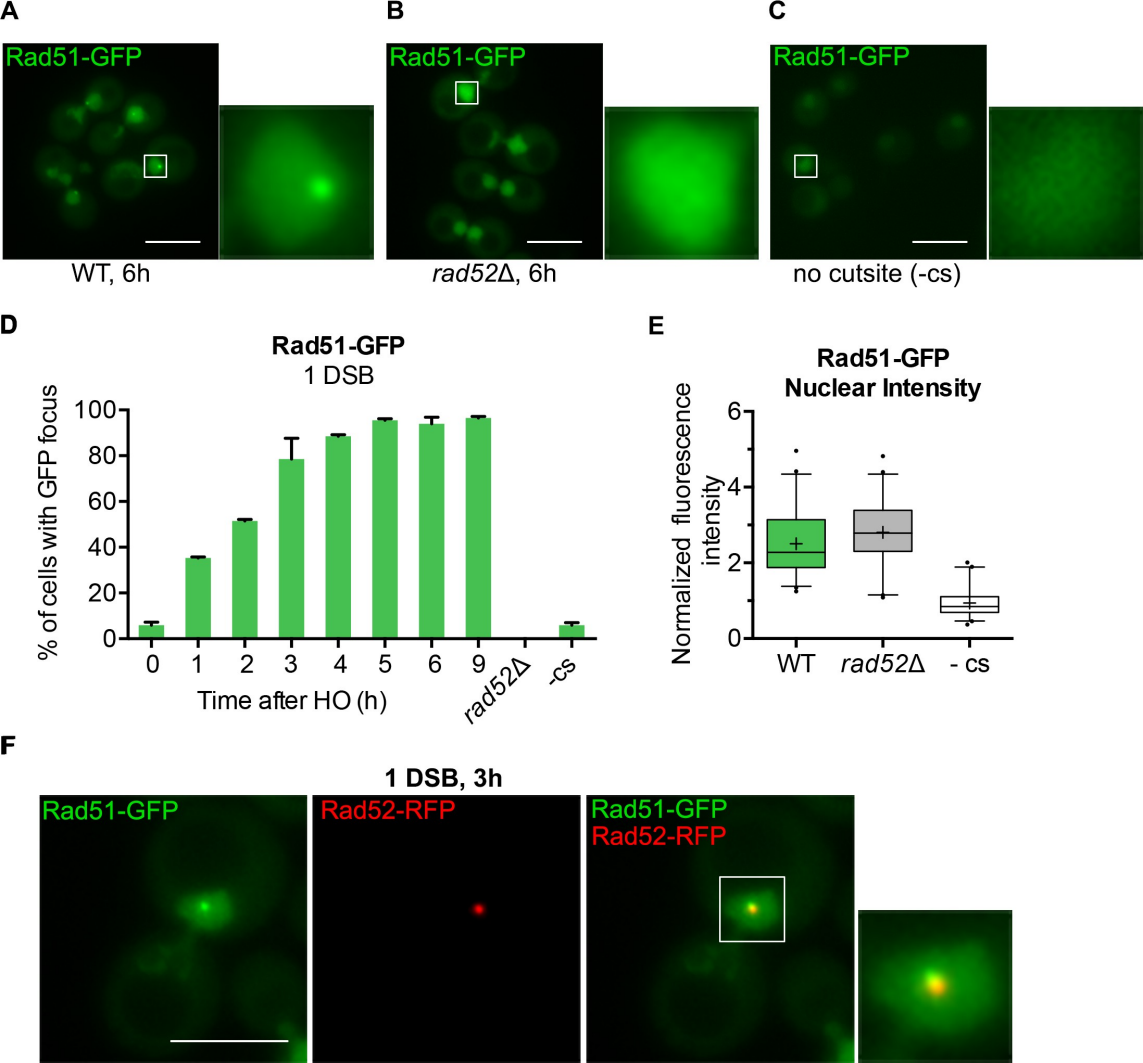
Rad51-GFP forms a DSB-dependent focus. **A)** Representative images of strain DW58 expressing Rad51-eGFP 6 h after HO induction. Magnification of white boxed nucleus shown to the right. Maximum projection of 10 z-stack images every 0.5 μm. Scale bar = 5 μm. **B)** Representative images of strain DW88 (*rad52*Δ) prepared as in (A). **C)** Representative images of strain DW94 (no HO cut site) prepared as in (A). **D)** Percentage of cells displaying Rad51-GFP foci in strain DW58, DW88, and DW94 at the indicated time or 3 h after HO induction (-cs and *rad52*Δ). Error bars represent the SD of three biological replicates of >150 cells per experiment/timepoint. **E)** Background-subtracted fluorescence intensities of nuclei in strains DW58 (WT) and DW88 (*rad52*Δ) 6 h after HO induction as described in (A). Box plots prepared as in Fig 1D. **F)** Representative image from strain DW89 expressing endogenous Rad51-eGFP and Rad52-RFP from its endogenous promoter on a centromeric containing plasmid 3 h after HO induction prepared as in (A).

An ideal tool for monitoring DSB formation and repair would be a fluorescent protein that performs a central role in homologous recombination. We created a Rad51-eGFP (GFP-S65T) fusion construct connected by a SSGSSG linker, which we have previously used to increase the functionality of other fusion proteins [48]. We integrated this fluorescent domain at the C-terminus of the genomic copy of *RAD51* in strain JKM179 in which a single, galactose-induced irreparable DSB, created by HO endonuclease, is induced within 30 minutes upon addition of galactose [55]. Rad51-eGFP is competent for adaptation whereas *rad51*Δ is adaptation-defective (S1E Fig), in agreement with previous findings [70]. In a strain suffering a single HO-induced DSB, more than 70% of cells displayed a single eGFP focus 3 h after inducing HO cleavage, increasing to >90% by 5 h (Figs 3A and 3D and S3A).

Because Rad51 filament formation is dependent on the Rad52 mediator, we confirmed that Rad51-eGFP foci were absent in *rad52*Δ cells (Figs 3B and 3D and S3B), as well as in cells lacking an HO cleavage site (Figs 3C and 3D and S3C). When Rad51-eGFP was co-expressed with Rad52-RFP, green and red foci colocalized (Figs 3F and S3D), as suggested from previous studies using chromosome spreads [32]. Rad51 abundance has been shown to increase after DNA damage [71, 72]. This increase is evident comparing the total nuclear intensity of Rad51-eGFP in cells with a DSB (with or without Rad52) compared to cells lacking the HO cleavage site (Fig 3E).

### Rad51-GFP cannot repair a DSB by homologous recombination in mitotic cells, but it is not dominant negative.

**Fig 4:**
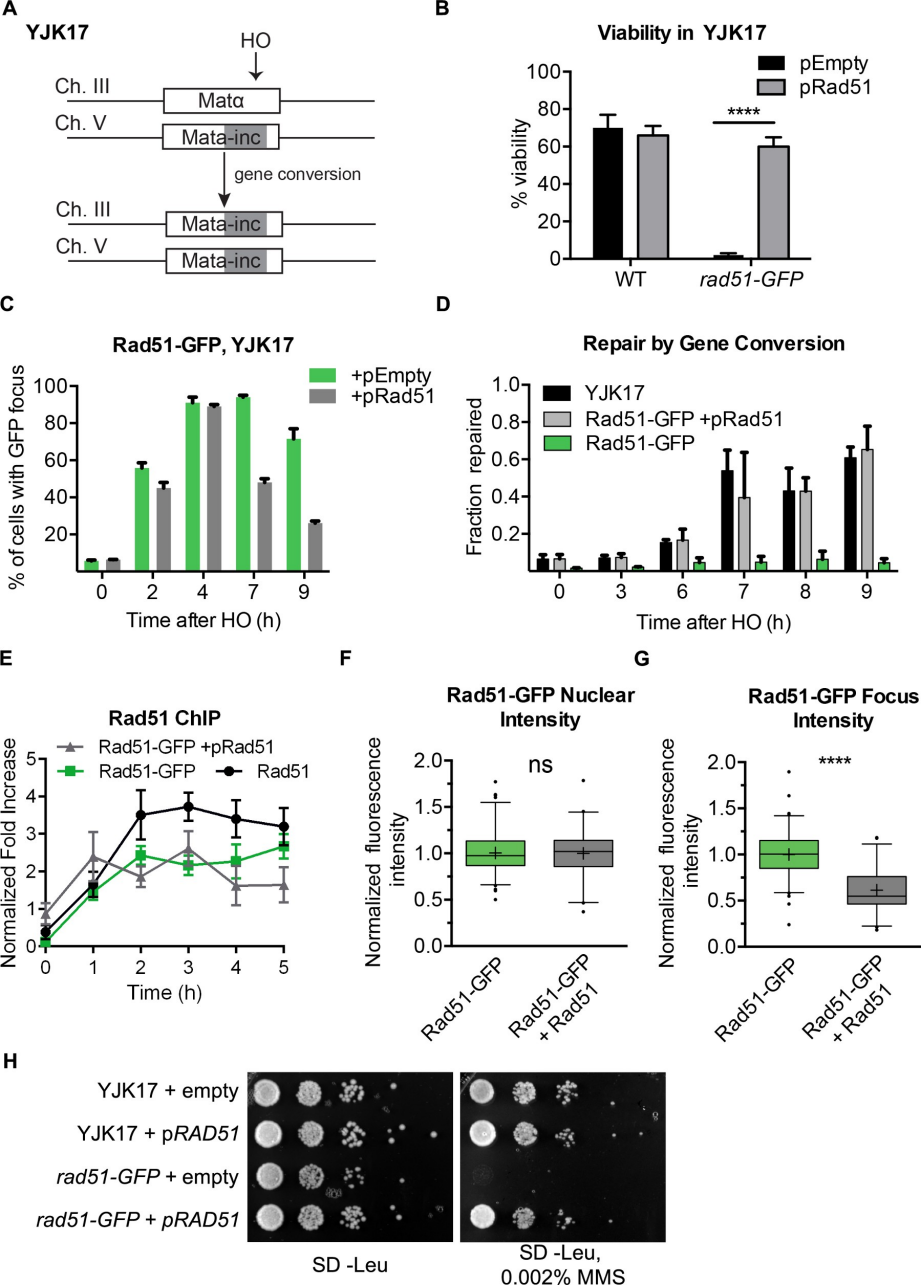
Rad51-GFP is not dominant negative. **A)** Schematic of repair by gene conversion in YJK17. An HO-induced DSB made on chromosome III is repaired by the *MAT****a***-inc donor sequence on chromosome V, which contains a mutation (-inc) that prevents further cutting. **B)** Percent viable cells following HO induction and repair through ectopic gene conversion in the indicated derivative of YJK17. Error bars represent the SD of three biological replicates. **C)** Percentage of cells displaying a focus in the indicated derivative of YJK17 at the indicated time. Error bars represent the SD of three biological replicates of >150 cells per experiment. **D)** qPCR analysis of the timing of DSB repair by gene conversion in the indicated derivatives of YJK17. **E)** Rad51 protein abundance ascertained by chromatin immunoprecipitation 250 bp distal to the HO cleavage site at *MAT*. Error bars represent SEM of three individual experiments performed in triplicate **F)** Fluorescence nuclear intensity of cells measured in (C) **G)** Measured intensity of the Rad51-GFP focus 3 h after HO induction in the indicated strain. Box and whisker plots prepared as in Fig 1D. **H)** Spot dilution assay without and with 0.002% MMS.

In the assays described thus far, DSBs were not repaired by HR because of the lack of a homologous donor template. To investigate the ability of Rad51-eGFP to participate in HR, we turned to strain YJK17, in which there is a DSB at *MAT*α on Chr3 and a single ectopic *MAT***a**-inc donor sequence inserted in Chr5 (Fig 4A) [47]. An HO break is repaired in roughly 80% of cells over the course of 6–9 h. YJK17 carrying Rad51-eGFP failed to repair the DSB (Fig 4B). Given the multimeric nature of the Rad51 filament and that many Rad51 mutations are dominant-negative [73, 74] we asked if Rad51-eGFP is dominant negative. We found that HO-induced recombination in strain YJK17 with Rad51-eGFP became repair-proficient after introducing wild type Rad51 on a centromeric plasmid, expressed from its own promoter (Fig 4B). The kinetics of repair, monitored by qPCR, were very similar for Rad51-GFP complemented by *RAD51* compared to wild type (Fig 4D). In parallel with repair, the percent of cells displaying a eGFP focus decreased from 80% at 4 h to ~50% by 7 h and fewer than 30% by 9 h, whereas without complementing Rad51, foci persisted (Fig 4C). This decrease correlated with the timing of repair, as monitored by qPCR (Fig 4D).

The ability of wild type Rad51 to complement Rad51-eGFP could also be seen by analyzing sensitivity to the DNA damaging agent, MMS. While Rad51-eGFP was indeed sensitive to MMS, this sensitivity was rescued by providing wild type *RAD51*, expressed from its own promoter on a centromere-containing plasmid (Fig 4H).

To test directly if Rad51-eGFP was bound to the DNA around the DSB, we performed chromatin immunoprecipitation using an antibody recognizing Rad51 to monitor Rad51-eGFP accumulation at the DSB induced at *MAT*α, as described previously [75]. Rad51-eGFP binding 250 bp from the DSB end increased over 2 h, reaching a plateau thereafter (Fig 4E). The rate and extent of Rad51-eGFP binding was very similar to wild type Rad51 for the first two h but leveled off at a lower value. That the extent of Rad51 binding was somewhat diminished when both Rad51-eGFP and Rad51 were expressed may indicate that the GFP derivative slightly impairs Rad51 filament formation, although recombination was proficient.

As a further measure of Rad51-eGFP binding, we compared the total nuclear fluorescence intensity and the accumulation of Rad51-eGFP in an HO endonuclease-induced focus. The total nuclear Rad51-eGFP signal was unaltered when wild type Rad51 was co-expressed (Fig 4F), but the intensity of fluorescence in the focus was reduced to about 50% of that seen in the absence of wild type protein (Fig 4G). This result suggests that Rad51-GFP is not strongly out-competed by wild type Rad51 protein in forming the filament that contains both wild type and Rad51-GFP monomers. Therefore, Rad51-eGFP effectively binds to resected DNA around a DSB and, when complemented with Rad51, will permit a detailed analysis of several steps in DSB repair.

### Rad51-GFP is competent in meiosis

**Fig 5:**
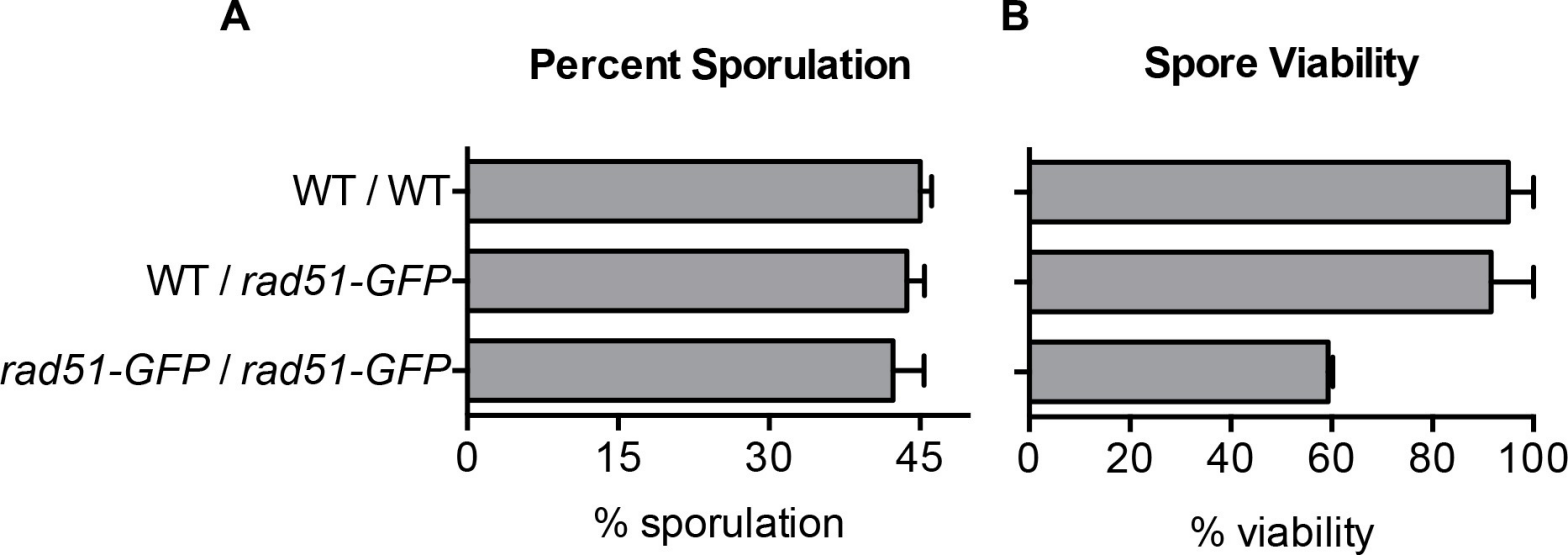
Rad51-GFP is competent in meiosis. **A)** Percent sporulated cells as determined by light microscopy in the indicated strain. Error bars represent the SD of three biological replicates of >150 cells per experiment. **B)** Quantification of spore viability after tetrad dissection of sporulated cells. Error bars are representative of three biological replicates of ≥24 tetrads dissected per experiment.

*Arabidopsis* Rad51-GFP proved to be meiosis-competent even though it failed to carry out mitotic recombination [35]. As noted above, this phenotype resembles a Rad51 “site II” mutation in budding yeast [36]. In meiosis, the critical functions of strand exchange depend on Rad51’s homolog, Dmc1, with Rad51 acting in an apparently allosteric fashion [36]. Nevertheless, Rad51 is required for efficient completion of interhomolog meiotic recombination; spore viability is only a few percent in the absence of Rad51 [71, 76]. We found that budding yeast Rad51-eGFP is meiosis-proficient. Diploids homozygous or heterozygous for Rad51-eGFP produced the same percentage of spores of as wild type. (Fig 5A). After tetrad dissection, spores resulting from diploids homozygous for Rad51-eGFP exhibited a 40% reduction in spore viability, but nevertheless 60% of spores were viable (Fig 5B). Thus, *S*. *cerevisiae* Rad51-GFP strongly resembles a site II mutation [36].

### Multiple DSBs form discrete Rad51-GFP foci

**Fig 6:**
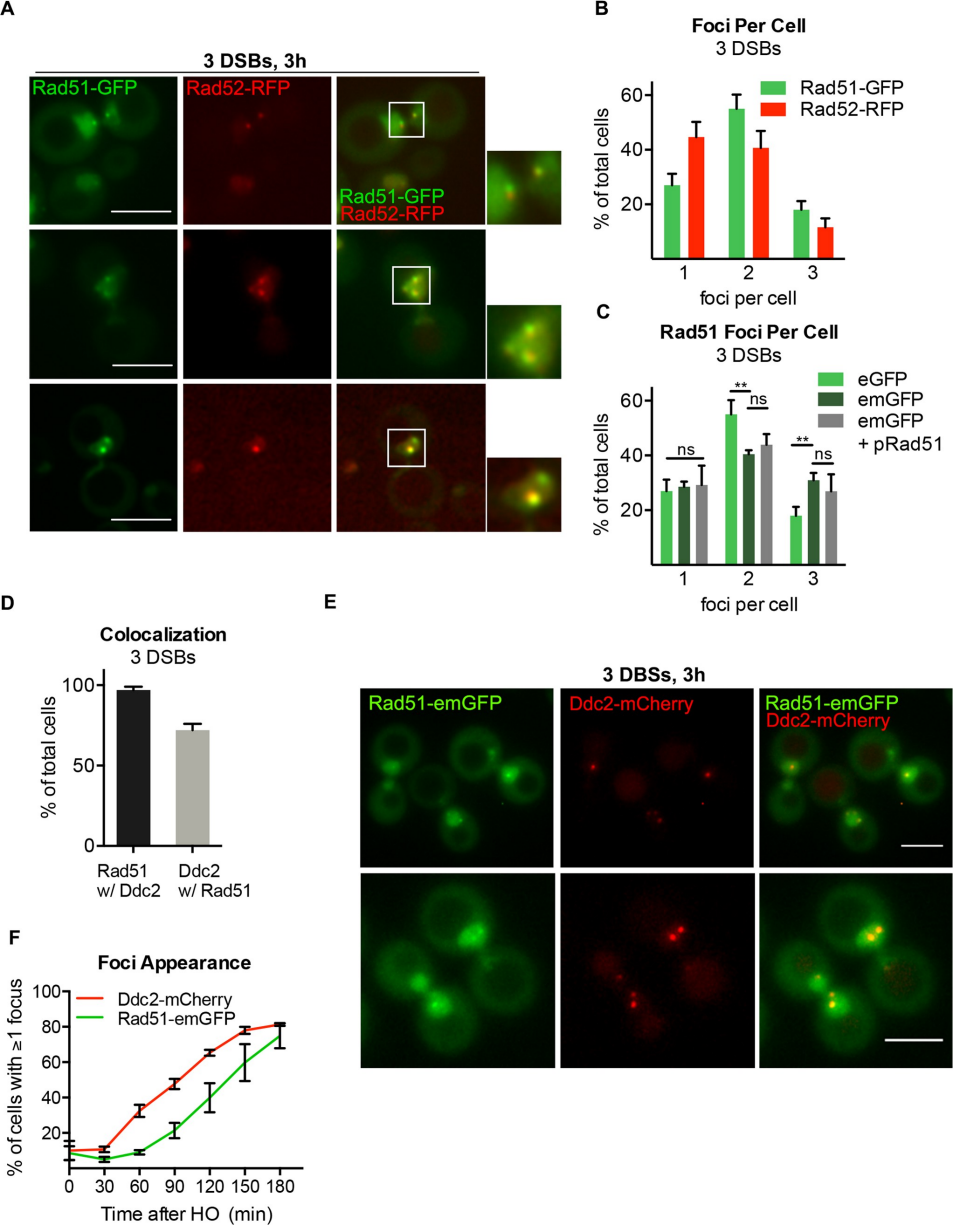
Rad51-GFP forms multiple foci in response to multiple DSBs. **A)** Representative images from strain DW106 expressing endogenous Rad51-eGFP and Rad52-RFP 3 h after HO induction. Magnification of white-boxed nucleus shown to the right. Maximum projection of 10–12 z-stack images every 0.5 μm. Scale bar = 5 μm. **B)** Rad51-GFP and Rad52-RFP foci in DW106 3 h after induction of HO. Error bars represent the SD of three biological replicates of >150 cells per experiment. **C)** Rad51-emGFP and Rad51-emGFP + pRad51 foci 3 h after HO induction. Rad51-eGFP data reproduced from (B) for comparison. Error bars presented as in (B). **D)** Percentage of Rad51-emGFP foci containing Ddc2-mCherry foci versus percentage of Ddc2-mCherry foci containing Rad51-emGFP foci 3h after HO induction. Error bars presented as in (B) **E)** Representative fluorescence images from cells expressing Ddc2-mCherry and Rad51-emGFP 3 h after HO induction in a 3 DSB strain. **F)** Percentage of cells displaying at least one Ddc2-mCherry or Rad51-emGFP focus at the indicated time after HO induction in a 3-DSB strain. Error bars presented as in (B).

We extended our analysis to monitor the localizations of several site-specific DSBs, to determine whether multiple DSBs would appear as a single Rad51-eGFP focus or as distinct foci, as we observed with Ddc2-GFP. We inserted Rad51-eGFP into strain YCSL004 carrying 3 HO cleavage sites. This strain also expressed Rad52-RFP from a centromere-containing plasmid, in addition to the wild type genomic *RAD52* [77–82]. Three h after HO induction, ~75% of cells were dumbbell-shaped, indicative of G_2_/M arrest (S4A Fig). We observed an average of two Rad51-eGFP foci (Fig 6A and 6B). This distribution was unchanged in *lig4*Δ cells (S4B Fig), in which repair by end-joining is blocked [83–85]. There were a number of instances where cells displayed a single Rad52-RFP focus but multiple Rad51-GFP foci (Figs 5A and 5B and S4C). In these cells, the single Rad52-RFP focus was typically large and always colocalized with one Rad51-GFP focus. Therefore, monitoring the number and locations of DSBs via Rad52 may underestimate the number of Rad51 foci in response to DSBs.

To avoid possible self-aggregation of Rad51-eGFP, we created a monomeric GFP (Rad51-emGFP) derivative. Three h after HO induction, we found that the percent of cells exhibiting a single focus was unchanged (Fig 6C). However, 15% fewer cells displayed 2 foci while 15% more displayed 3 foci when compared to Rad51-eGFP (Fig 6C). This distribution was unchanged after expressing a wild type copy of Rad51 from a centromere-containing plasmid (Fig 6C)

We then monitored the localization of Ddc2-mCherry and Rad51-emGFP co-expressed in our 3-break system, 3 h after HO induction, to compare the localization profiles of both DSB markers. Nearly 100% of Rad51-emGFP foci colocalized with Ddc2-mCherry foci (Fig 6D and 6E). In contrast, about 30% of Ddc2-mCherry foci lacked Rad51-emGFP (Fig 6D and 6E). When we monitored Ddc2-mCherry and Rad51-emGFP foci simultaneously at 30-min intervals after HO induction in cycling cells we found that, within 60 min, nearly 40% of cells displayed at least one Ddc2-mCherry focus while Rad51-emGFP was present in only ~5% of cells. By 120 min, this difference was still apparent, in that ~60% of cells displayed ≥ one Ddc2-mCherry focus while only ~30% displayed Rad51-emGFP foci (Fig 6F). However, three h after HO induction, ≥1 Ddc2 and Rad51 foci were present in an equal number of cells, although–as noted above–some Ddc2 foci did not exhibit a Rad51 focus. These data support the proposal that checkpoint activation precedes the loading of recombination machinery [40].

## Discussion

DSB repair must be coordinated in space and time in order to faithfully repair lesions to the genome. The roles of many proteins involved in DSB repair have been elucidated through *in vitro* and *in vivo* biochemistry, but the lack of suitable live-cell markers in budding yeast has provided a barrier to studying DSB repair in real-time. Here, we report DSB dynamics in single- and multiple-break conditions using two different fluorescently tagged proteins that carry out different functions in response to DNA damage; the recombinase Rad51-GFP and the checkpoint-related protein Ddc2-GFP. In both cases, multiple DSBs usually resulted in multiple fluorescent foci.

Using Rad51-GFP or Ddc2-GFP in our 3-DSB system, the majority of cells exhibit two or three foci. Rad51-GFP foci usually colocalize with Rad52-RFP, but there are a significant number of cells with more GFP foci than RFP foci. Previous studies have looked specifically at the role of Rad52 in organizing a “repair center” yeast [29, 86]. Our data suggest that monitoring Rad52 focus formation may underestimate the number of DSBs throughout the nucleus. This difference may in part reflect the temporal recruitment of DSB repair proteins to the site of DSBs such that the continued presence of Rad52 at a DSB may not be necessary once a Rad51 filament has been established; however our previous analysis of nuclear spreads by immunofluorescence suggested that Rad52 which plays a Rad51-dependent role in completing gene conversion [30] persisted longer than Rad51 [32].

To test whether Rad52 recruits multiple DSBs into a common locus, we used Ddc2-GFP, which forms foci independent of Rad52. In our 3-DSB strain, we see an average of 2 Ddc2-GFP foci per cell, but still about 25% of cells display a single focus, as with Rad51-GFP. However, this distribution remains unchanged in a *rad52*Δ derivative. Furthermore, using live cell imaging in a strain with 3 DSBs, we found that Ddc2-coated DSBs are often highly dynamic; a single focus can split into multiple foci while multiple foci may coalesce into one focus. Again, this behavior was unchanged in a *rad52*Δ derivative. We also examined the role of microtubules in organizing DSBs as previous studies have implicated microtubules in promoting chromatin motion [20, 23, 65, 66], but found that depolymerizing microtubules did not alter the behavior or number of apparent DSBs. Therefore, we conclude that Rad52 and microtubules are not required for organizing multiple DSBs into one specific nuclear location.

While our Rad51-GFP construct is not able, by itself, to repair DSBs by homologous recombination, it is not dominant-negative in mitotic cells and supports recombination in meiosis. Biochemical analysis of human Rad51 fused to GFP determined that the fluorescent tag prevented Rad51 from engaging in the pairing of homologous sequences by inhibiting double-stranded DNA from binding in Rad51’s secondary DNA binding site (site II) [38]. We envision the same to be true of our Rad51-GFP construct because our ChIP experiments and microscopy suggest that Rad51-GFP can efficiently bind to ssDNA and form a filament, its first step in homologous recombination. However, when Rad51-GFP is the sole copy of Rad51 in cells, DSB repair by homologous recombination is incomplete, presumably at the strand exchange step.

Rad51-GFP’s defect in ectopic gene conversion and in MMS-resistance is suppressed by addition of a single second copy of wild type Rad51 expressed from its endogenous promoter. However, it is not that wild type Rad51 simply excludes Rad51-GFP from binding ssDNA, since Rad51-GFP readily forms a DSB-dependent focus in the presence of wild type Rad51, similar to a Rad51-CFP in fission yeast [39]. About half of the Rad51 monomers in the focus appear to be GFP-tagged. Our data suggest either that a functional Rad51 filament does not require every Rad51 molecule to be functional or that subunit-subunit interactions between wild type and GFP-tagged Rad51 correct the defect. The exact stoichiometry for a functional filament cannot be determined from these experiments, but from previous analysis of the minimum requirements Rad51-mediated strand exchange *in vitro* [87, 88] and *in vivo* [45], it is likely that there need to be at least two to three functional Rad51 molecules in tandem to facilitate minimal Rad51-mediated strand exchange.

Increased chromatin motion in response to a DSB is believed to aid in DNA repair through facilitating in homology search throughout the genome [12, 62–64]. but the precise mechanism for this motion is unclear [89]. Our characterizations of these live-cell markers of DSBs will facilitate a more detailed study of the the motions of broken chromosomes.

## Supporting information

S1 Fig Ddc2-GFP localization in multi-break strains. **A)** Representative full field image of strain VE290 expressing Ddc2-GFP 3 h after HO induction. **B)** Representative full field image of strain DW546 (*rad52*Δ) expressing Ddc2-GFP 3 h after HO induction. **C)** Representative full field image of strain VE290 expressing Ddc2-GFP in logarithmically growing cells. **D)** Identical to C) but for a *rad52*Δ derivative. Maximum projection of 10–12 z-stack images every 0.5 μm. Scale bar = 5 μm. **E)** Percentage of cells adapting to a single DSB at either 8 or 24 h after HO induction. Error bars represent SD of three experiments. 150 cells observed in total.
(TIF)

S2 Fig Ddc2-GFP and Mps3-mCherry localization without and with nocodazole. **A)** Representative full field image of Ddc2-emGFP and Mps3-mCherry 3 h after HO induction and 10 min after DMSO (vehicle) addition. Maximum projection of 12 z-stack images every 0.5 μm. Scale bar 5 μm. **B)** Representative full field image of Ddc2-emGFP and Mps3-mCherry 3 h after HO induction and 10 min after 15 μg/ml nocoadazole addition. Images prepared as in (A).
(TIF)

S3 Fig Rad51-GFP localization. **A)** Representative full field image of strain DW58 expressing endogenous Rad51-GFP 6 h after HO induction. **B)** Representative full field image of strain DW88 (*rad52*Δ) expressing Rad51-eGFP 6 h after HO induction. **C)** Representative images of strain DW94 (no HO cut site) expressing Rad51-eGFP 6 h after HO induction. **D)** Representative full field image from strain DW89 expressing endogenous Rad51-eGFP and Rad52-RFP from its endogenous promoter on a low copy plasmid 3 h after HO induction. Maximum projection of 10–12 z-stack images every 0.5 μm. Scale bar = 5 μm.
(TIF)

S4 Fig Rad51-GFP localization in multi-break strains. **A)** Cell morphology at the indicated time after HO induction. Data represent the average of three individual experiments observing >150 cells per experiment. **B)** Rad51-GFP foci in strain DW123 (*lig4*Δ). **C**) Representative full field images of strain DW106 expressing Rad51-eGFP and Rad52-RFP 3 h after HO induction.
(TIF)

S1 Movie Ddc2-GFP in 3 DSB strain DW546 (*rad52*Δ) 3 h after HO induction. Scale bar = 5 μm.
(AVI)

S2 Movie Ddc2-GFP in 3 DSB strain DW546 (*rad52*Δ) 3 h after HO induction. Scale bar = 5 μm.
(AVI)

S3 Movie Ddc2-GFP in 3 DSB strain DW546 (*rad52*Δ) 3 h after HO induction. Scale bar = 5 μm.
(AVI)

S4 Movie Ddc2-GFP in 3 DSB strain VE290 3 h after HO induction. Scale bar = 5 μm.
(AVI)

S5 Movie Ddc2-GFP in 3 DSB strain VE290 3 h after HO induction. Scale bar = 5 μm.
(AVI)

S6 Movie Ddc2-GFP in 3 DSB strain VE290 3 h after HO induction. Scale bar = 5 μm.
(AVI)

S7 Movie Ddc2-GFP in 3 DSB strain VE290 3 h after HO induction. Scale bar = 5 μm.
(AVI)

S8 Movie Ddc2-GFP in 3 DSB strain VE290 3 h after HO induction. Scale bar = 5 μm.
(AVI)

S9 Movie Ddc2-GFP in 3 DSB strain VE290 3 h after HO induction. Scale bar = 5 μm.
(AVI)

S10 Movie Ddc2-GFP in 3 DSB strain VE290 3 h after HO induction. Scale bar = 5 μm.
(AVI)

S11 Movie Ddc2-GFP in 3 DSB strain VE290 3 h after HO induction. Scale bar = 5 μm.
(AVI)

S12 Movie Ddc2-GFP in 3 DSB strain VE290 3 h after HO induction. Scale bar = 5 μm.
(AVI)

S13 Movie Ddc2-GFP in 3 DSB strain VE290 3 h after HO induction. Scale bar = 5 μm.
(AVI)

S14 Movie Ddc2-GFP in 3 DSB strain VE290 3 h after HO induction. Scale bar = 5 μm.
(AVI)

S1 Table Strains used in this study. (DOCX)

S2 Table Primers used in this study. (DOCX)

S3 Table Plasmids used in this study. (DOCX)
