# Supplementary figures and images for: Live cell monitoring of double strand breaks in S. cerevisiae
Source: PLoS Genet. 2019 Mar 1;15(3):e1008001. doi: 10.1371/journal.pgen.1008001 (PMC6415866; doi:10.1371/journal.pgen.1008001)

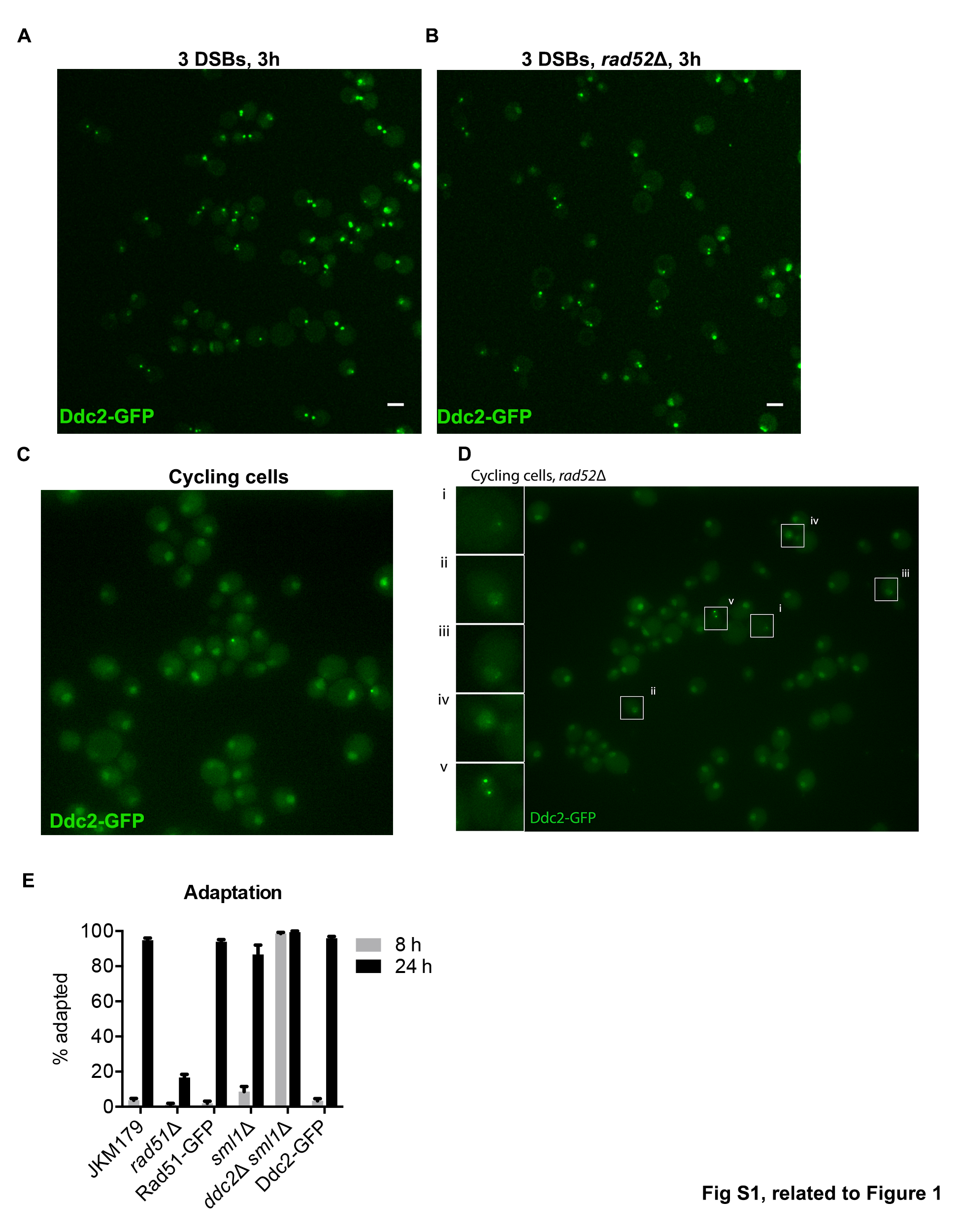

Supplement: S1 Fig — A) Representative full field image of strain VE290 expressing Ddc2-GFP 3 h after HO induction. B) Representative full field image of strain DW546 (rad52Δ) expressing Ddc2-GFP 3 h after HO induction. C) Representative full field image of strain VE290 expressing Ddc2-GFP in logarithmically growing cells. D) Identical to C) but for a rad52Δ derivative. Maximum projection of 10–12 z-stack images every 0.5 μm. Scale bar = 5 μm. E) Percentage of cells adapting to a single DSB at either 8 or 24 h after HO induction. Error bars represent SD of three experiments. 150 cells observed in total. (TIF) [file pgen.1008001.s001.tif]

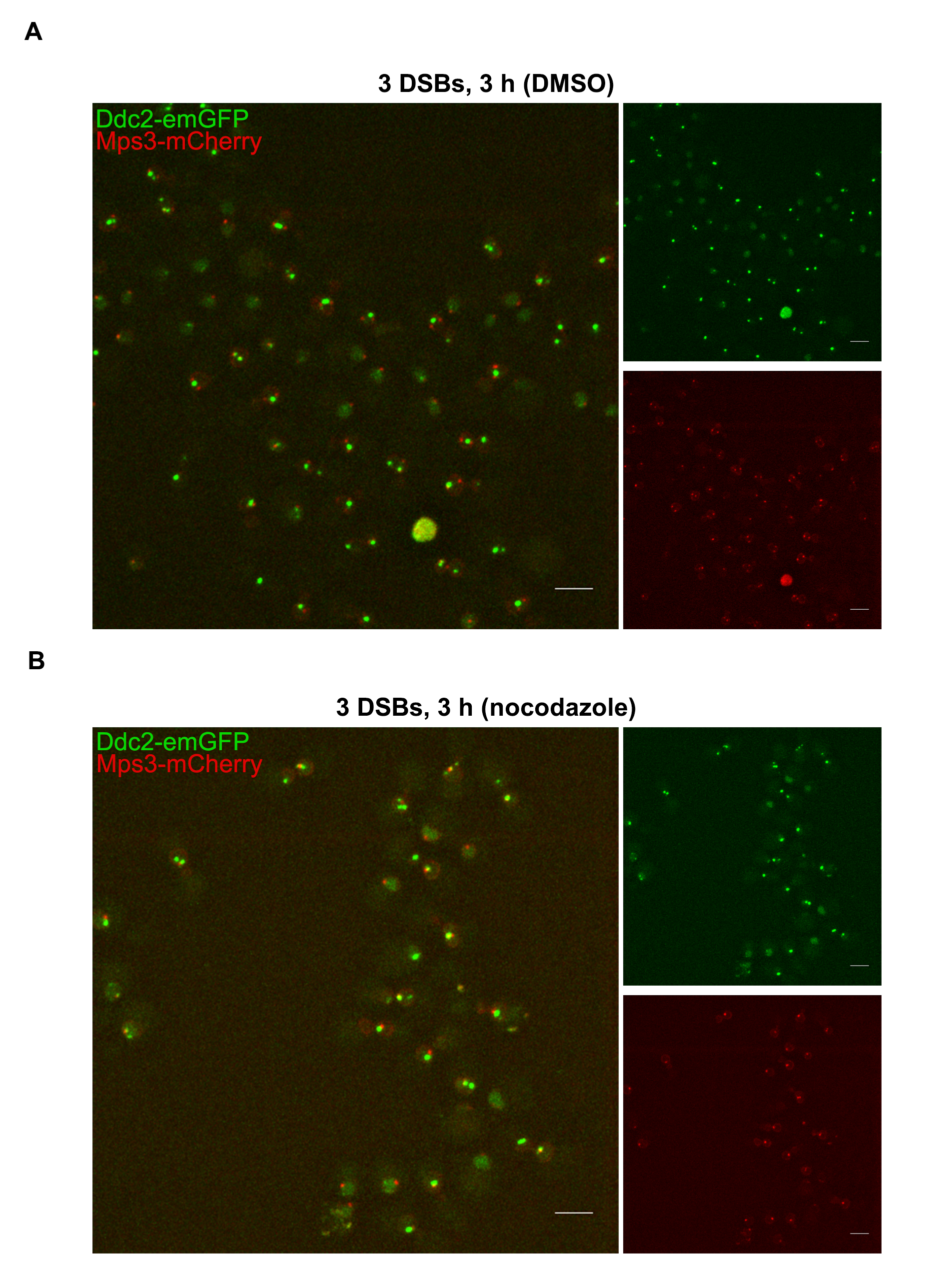

Supplement: S2 Fig — A) Representative full field image of Ddc2-emGFP and Mps3-mCherry 3 h after HO induction and 10 min after DMSO (vehicle) addition. Maximum projection of 12 z-stack images every 0.5 μm. Scale bar 5 μm. B) Representative full field image of Ddc2-emGFP and Mps3-mCherry 3 h after HO induction and 10 min after 15 μg/ml nocoadazole addition. Images prepared as in (A). (TIF) [file pgen.1008001.s002.tif]

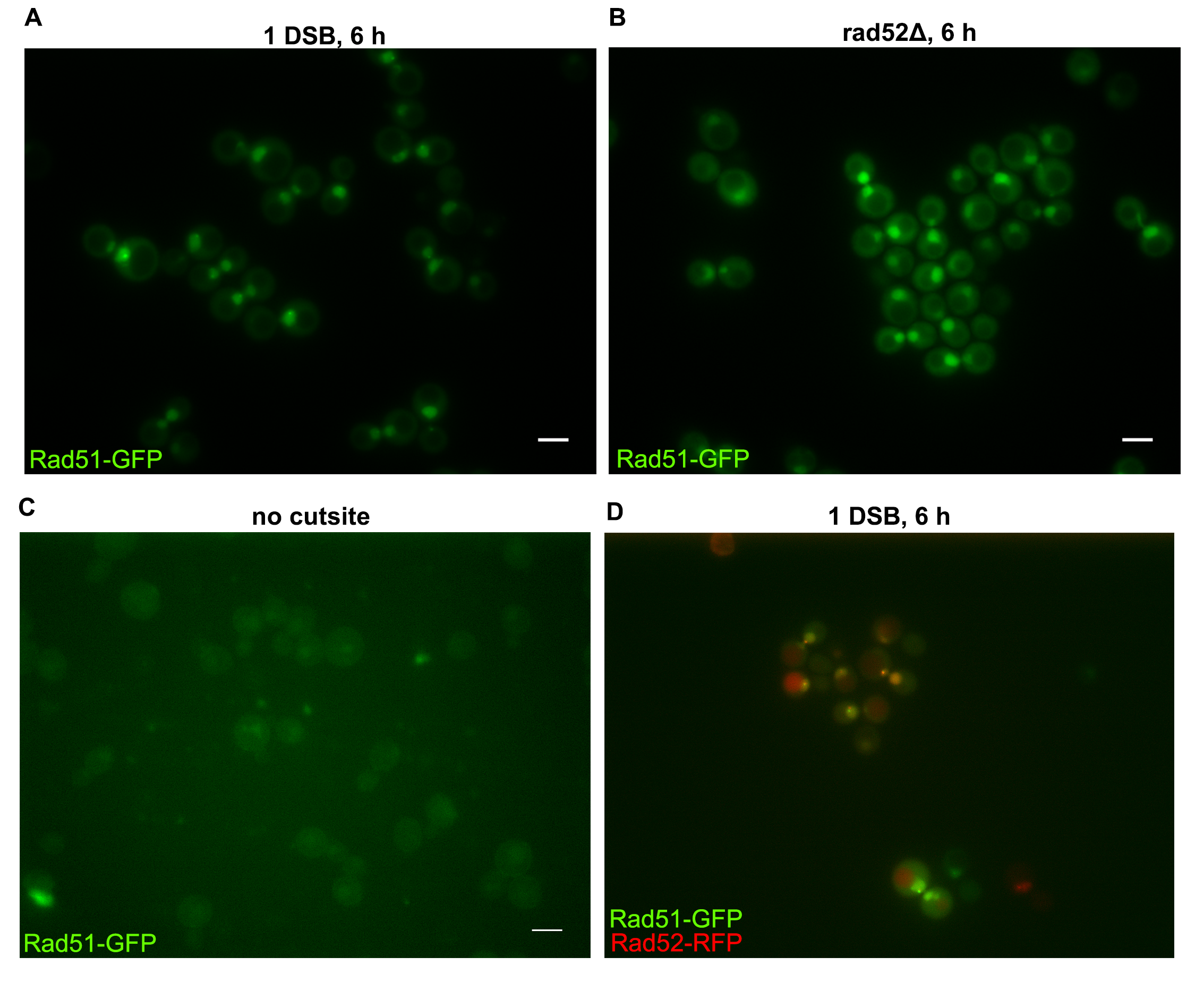

Supplement: S3 Fig — A) Representative full field image of strain DW58 expressing endogenous Rad51-GFP 6 h after HO induction. B) Representative full field image of strain DW88 (rad52Δ) expressing Rad51-eGFP 6 h after HO induction. C) Representative images of strain DW94 (no HO cut site) expressing Rad51-eGFP 6 h after HO induction. D) Representative full field image from strain DW89 expressing endogenous Rad51-eGFP and Rad52-RFP from its endogenous promoter on a low copy plasmid 3 h after HO induction. Maximum projection of 10–12 z-stack images every 0.5 μm. Scale bar = 5 μm. (TIF) [file pgen.1008001.s003.tif]

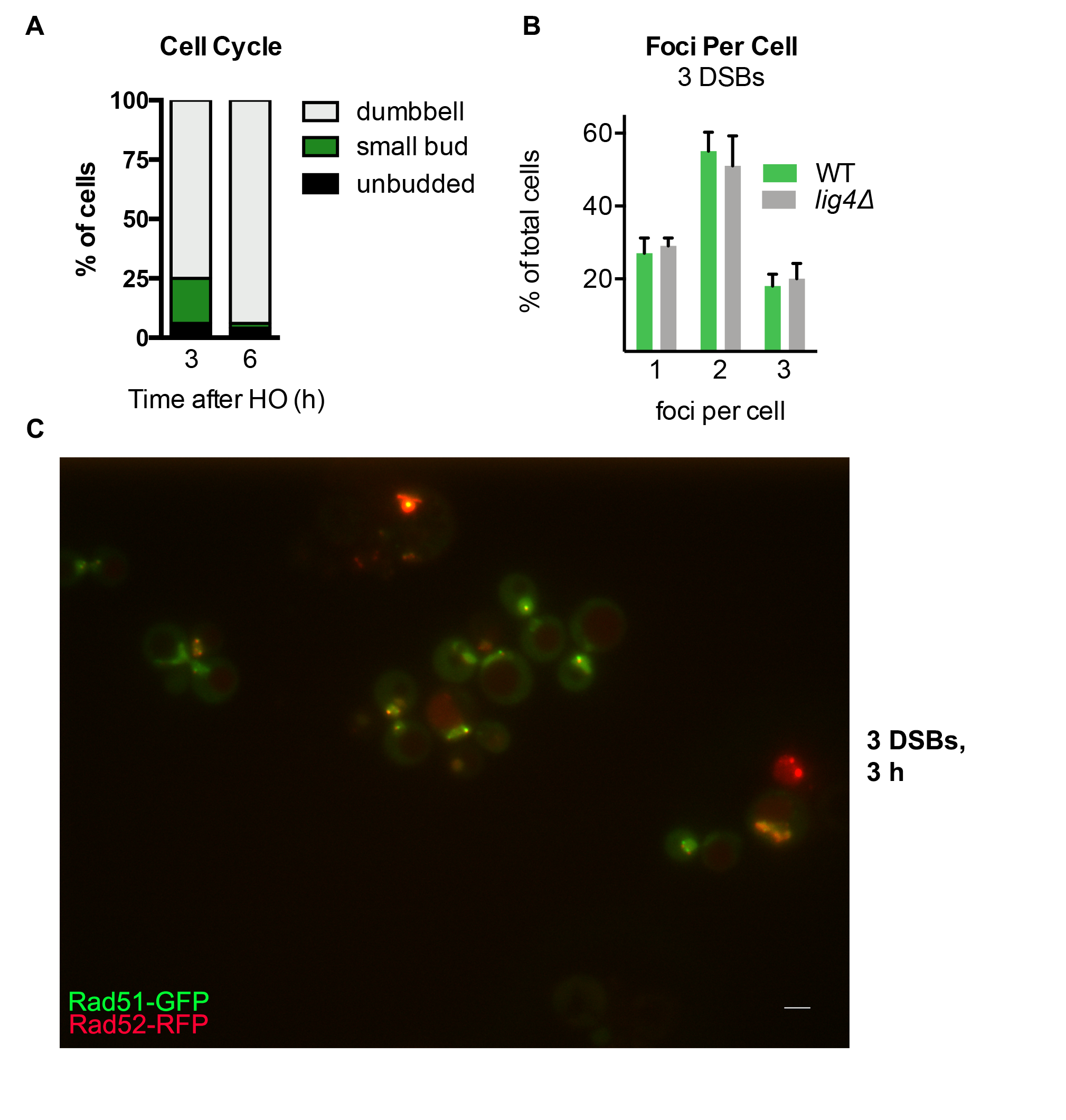

Supplement: S4 Fig — A) Cell morphology at the indicated time after HO induction. Data represent the average of three individual experiments observing >150 cells per experiment. B) Rad51-GFP foci in strain DW123 (lig4Δ). C) Representative full field images of strain DW106 expressing Rad51-eGFP and Rad52-RFP 3 h after HO induction. (TIF) [file pgen.1008001.s004.tif]
