# Supplementary material for: Live cell monitoring of double strand breaks in S. cerevisiae
Source: PLoS Genet. 2019 Mar 1;15(3):e1008001. doi: 10.1371/journal.pgen.1008001 (PMC6415866; doi:10.1371/journal.pgen.1008001)
Supplement: S1 Table — (DOCX) [file pgen.1008001.s019.docx]

**S19 Table. Strains used in this study**

| **Strain** | **Genotype** | **Parent Strain** | **Reference** | **Figures** |
| --- | --- | --- | --- | --- |
| JKM179 | hoΔ hmlΔ::ADE1 MATα hmrΔ::ADE1 ade1-100 leu2-3,112 lys5 trp1::hisG ura3-52 ade3::GAL10::HO | Background | ([46](#_ENREF_46)) | S1E |
| YCSL004 | hoΔ hmlΔ::ADE1 MATα hmrΔ::ADE1 ade1-100 leu2-3,112 lys5 trp1::hisG ura3-52 ade3::GAL10::HO Chr6: 97749 nt::HPH:HOcs Chr2: 252kb::HOcs-URA3 | Background | ([50](#_ENREF_50)) |  |
| YJK17 | MAT*α*ho*Δ*hml*Δ::*ADE1 hmr*Δ::*ADE1 arg5,6*Δ::*HPH*::*MAT***a****-inc*ade1*-*100 leu2,3*-*112 lys5 trp*::*hisG ura3*-*52 ade3*::*GAL*::*HO | Background | ([66](#_ENREF_66)) | 4A, 4D, 4E |
| LSY1228 | Mata leu2-3,112 trp1-1 ura3-1 can1-100 ade2-1 his3-11,15 RAD5 *6 Ty1-HOcs-*HIS3 | Background | ([90](#_ENREF_90)) |  |
| VE290 | *Ddc2-GGSGGS-eGFP::TRP1* | YCSL004 | This study | 1A – 1D, S1E, S1A |
| DW52 | Rad51-GGSGGS-eGFP::TRP1 | YJK17 | This study |  |
| DW58 | Rad51-GGSGGS-eGFP::TRP1 | JKM179 | This study | 3A, 3D, 3E, S3A, S1E |
| DW88 | Rad51-GGSGGS-eGFP::TRP1 rad52::KAN | JKM179 | This study | 3B, 3D, 3E, S3B |
| DW89 | Rad51-GGSGGS-eGFP::TRP1 + pRad52-RFP (LEU2) | JKM179 | This study | 3F, S3D |
| DW94 | Rad51-GGSGGS-eGFP::TRP1 -HOcs | JKM179 | This study | 3C – 3E, S3C |
| DW106 | *rad51-GGSGGS-GFP::TRP1* *+pRad52-RFP(LEU2)* | YCSL004 | This study | 6A, 6B, S4C |
| DW539 | Rad51-GGSGGS-eGFP::TRP1 +pRS315(LEU2) | YJK17 | This study | 4B – 4H |
| DW540 | Rad51-GGSGGS-eGFP::TRP1 +pRad51(LEU2) | YJK17 | This study | 4B – 4H |
| DW553 | pRS315(LEU2) | YJK17 | This study | 4B, 4D, 4E, 4H |
| DW546 | ddc2-GGSGGS-eGFP::TRP1 rad52::KAN | YCSL004 | This study | 1B – 1D, S1B |
| W303-1A | MAT**a** leu2-3,112 trp1-1 can1-100 ura3-1 ade2-1 his3-11,15 | Background | ([91](#_ENREF_91)) | 5A, 5B |
| W303-1B | MATα leu2-3,112 trp1-1 can1-100 ura3-1 ade2-1 his3-11,15 | Background | ([91](#_ENREF_91)) | 5A, 5B |
| DW504 | *rad51-GGSGGS-eGFP::TRP1* | W303-1A | This study | 5A, 5B |
| DW505 | *rad51-GGSGGS-eGFP::KAN* | W303-1B | This study | 5A, 5B |
| DW554 | MATα/a *rad51-GGSGGS-GFP::KAN/rad51-GGSGGS-GFP::TRP1* | W303 | This study | 5A, 5B |
| DW123 | *rad51-GGSGGS-eGFP::TRP1 lig4::NAT* | YCSL004 | This study | S4B |
| DW593 | *Rad51-GGSGGS-eGFPA206K::TRP1 (emGFP)* | YCSL004 | This study | 6C – 6F |
| DW594 | *Ddc2-GGSGGS-eGFPA206K::TRP1 (emGFP)* | YCSL004 | This study | 1B, 1C, 2A – 2D |
| DW594 | *Ddc2-GGSGGS-eGFPA206K::TRP1 (emGFP) rad52::KAN* | YCSL004 | This study | 1B, S1D |
| DW613 | *Rad51-GGSGGS-eGFPA206K::TRP1 (emGFP) Ddc2-mCherry::KAN* | YCSL004 | This study | 6D – 6F |
| DW619 | *Rad51-GGSGGS-eGFPA206K::TRP1 (emGFP) + pRad51* | YCSL004 | This study | 6C |
| GM42 | *Ddc2-eGFP::TRP1* | JKM179 | This study | S1E |
| DW65 | *rad51::HPH* | JKM179 | This study | S1E |
| YFD756 | *sml1::KAN* | JKM179 | ([49](#_ENREF_49)) | S1E |
| YFD192 | *sml1::KAN ddc2::HPH* | JKM179 | ([49](#_ENREF_49)) | S1E |
