# Supplementary material for: Live cell monitoring of double strand breaks in S. cerevisiae
Source: PLoS Genet. 2019 Mar 1;15(3):e1008001. doi: 10.1371/journal.pgen.1008001 (PMC6415866; doi:10.1371/journal.pgen.1008001)
Supplement: S2 Table — (DOCX) [file pgen.1008001.s020.docx]

**S20 Table. Primers used in this study**

| **Primer Name** | **Sequence** | **Use** |
| --- | --- | --- |
| VE085 Ddc2-GFP For | ATCTAACCACACTAGAGGAGGCCGATTCATTATATATCTCAATGGGACTG*GGTGGTTCCGGTGGTTCC*CGGATCCCCGGGTTAATTAA | C terminal GFP tagging of Ddc2 (forward) |
| VE086 Ddc2-GFP Rev | ATTACAAGGTTTCTATAAAGCGTTGACATTTTCCCCTTTTGATTGTTGCCGAATTCGAGCTCGTTTAAAC | C terminal GFP tagging of Ddc2 (reverse) |
| DW060 Rad51-GFP 2F | CTATGAAGATGGTGTTGGTGACCCCAGAGAAGAAGACGAG*GGTGGTTCCGGTGGTTCC*CGGATCCCCGGGTTAATTAA | C terminal GFP tagging of Rad51 (forward) |
| DW063 Rad51-GFP 1R | GAAAGTAAACCTGTGTAAATAAATAGAGACAAGAGACCAAATACGAATTCGAGCTCGTTTAAAC | C terminal GFP tagging of Rad51 (reverse) |
| RAD51HPH1 | ATGTCTCAAGTTCAAGAACAACATATATCAGAGTCACAGCTTCAGTACGGGCATAGGCCACTAGTGGATCTG | Genomic deletion of RAD51 with HPH (forward) |
| RAD51HPH1 | CTACTCGTCTTCTTCTCTGGGGTCACCAACACCATCTTCATAGATCGCGATTTCAGCTGAAGCTTCGTACGC | Genomic deletion of RAD51 with HPH (reverse) |
| DW091 Rad52 1F | GGAGGTTGCCAAGAACTGCTGAAGGTTCTGGTGGCTTTGGTGTGTTGTTGATGCGTACGCTGCAGGTCGAC | Genomic deletion of RAD52 with KAN (forward) |
| DW092 Rad52 1R | AGTAATAAATAATGATGCAAATTTTTTATTTGTTTCGGCCAGGAAGCGTTTCAATCGATGAATTCGAGCTCG | Genomic deletion of RAD52 with KAN (reverse) |
| DW493 mps3GFP 1F | CATCCCGCTTCTAACGTCCCATCATTTGGCCAAGATGAGCTAGATCAACGGATCCCCGGGTTAATTAA | C terminally tag mCherry tag Mps3 (forward) |
| DW494 mps3GFP 1R | CGATTTTCTGGGGGCCAGGGGGTTAGAACGTTTAATTTTTTATTGTCGTGAATTCGAGCTCGTTTAAAC | C terminally tag mCherry tag Mps3 (reverse) |
| DW265 Rad51 3F | CCGTAGTTTCCATATACTAGTAGTTGAG | Confirm deletion / GFP tagging (forward) |
| DW266 Rad51 3R | AGATAAAAATGTACGGAACGCAACC | Confirm deletion / GFP tagging (reverse) |
| ddc2-400 FP | CGTATTGTGTGGCACCGATGTTAAGCAC | Confirm GFP tagging (forward) |
| ddc2+400 RP | CTCACACCTTGTGTAACAGATGTGGTCG | Confirm GFP tagging (reverse) |
| DW093 Rad52 2F | CCTGTAATGTCCTTTCGTCTTC | Confirm deletion (forward) |
| DW094 Rad52 2R | CGACACATGGAGGAAAGAAAA | Confirm deletion (reverse) |
| MATp13 | GTTAAGATAAGAACAAACAAgGATGCT | Monitor repair by qPCR Chr3 201210-201183 |
| MATYp4 | GATCTAAATAAATTCGTTTTCAATGATTAAAATAG | Monitor repair by qPCR Chr3 294342-294342 |
| CSL177-Yalpha p1 | CTCACAGTTTGGCTCCGGTG | Chr3 200750-200769 |
| NS047-Slx4p7 | ACCACTAAGTGACAAAGAACTACG | Chr12 413147-413124 Crick R-L |
| Slx4p1 | GATATGGACCTCTGTCCTTCCT | Chr12 412956-412977 Watson L->R |
| DW548 | GATGGCCCTGTCCTTTTACCAGACAAtCATTACCTGTCCACACAATCTaaaCTTTCGAAAGATCCCAACGAAAAGAGAGACC | SSTR oligo to mutate A206 to K in eGFP sequence. Also disrupts PAM |
| DW549 | TTACCTGTCCACACAATCTGGATCA | gRNA oligo to clone into bRA89 at BplI site. Directs Cas9 to cut within the eGFP sequence. |
| DW550 | CAGATTGTGTGGACAGGTAAGTTTT | gRNA oligo to clone into bRA89 at BplI site. Duplex with oDW549. Directs Cas9 to cut within the eGFP sequence. |
| MT101 +50 | CGCTTCTTTGGCCAATGC | Sense oligo 50 bp into *PHO5* ORF. For ChIP control |
| MT102 +100 | GGGTACCAATCTTGTCGAC | Antisense oligo 100 bp in *PHO5* ORF. For ChIP control |
| MATYαP5 | CATACAGAAACACAGCGG | Sense oligo localed in MATY alpha sequence. For Rad51 ChIP at MAT |
| MATYαP6 | AGGAAGGAACAGGAATCTGG | Antisense oligo localed in MATY alpha sequence. For Rad51 ChIP at MAT |
