# Supplementary material for: Live cell monitoring of double strand breaks in S. cerevisiae
Source: PLoS Genet. 2019 Mar 1;15(3):e1008001. doi: 10.1371/journal.pgen.1008001 (PMC6415866; doi:10.1371/journal.pgen.1008001)
Supplement: S3 Table — (DOCX) [file pgen.1008001.s021.docx]

**S21 Table. Plasmids used in this study**

| **Plasmid Name** | **Relevant Information** | **Alias** | **Citation** |
| --- | --- | --- | --- |
| pRS315 | CEN6/ARS4 *LEU2* | pEmpty | ([92](#_ENREF_92)) |
| bRA89 | pPGK1-Cas9 *HPH* | bRA89 | ([83](#_ENREF_83)) |
| pIG02 | pRad52-RFP *CEN6/ARS4* *LEU2* | pRad52-RFP | M. Lisby |
| pRS315-Rad51 | pRad51 *CEN6/ARS4* *LEU2* | pRad51 | ([94](#_ENREF_94)) |
| pDW54 | pPGK1-Cas9 *HPH* gRNA targeting eGFP |  | This study |
